# Precision Healthcare of Type 2 Diabetic Patients Through Implementation of Haptoglobin Genotyping

**DOI:** 10.3389/fcvm.2018.00141

**Published:** 2018-10-16

**Authors:** Bradley F. Bale, Amy L. Doneen, David J. Vigerust

**Affiliations:** ^1^Washington State University Elson S. Floyd College of Medicine, Spokane, WA, United States; ^2^Vanderbilt University School of Medicine, Nashville, TN, United States; ^3^MyGenetx Clinical Laboratory, Franklin, TN, United States

**Keywords:** haptoglobin, diabetes, stroke, cardiovascular disease, inflammation, precision healthcare, genetics, vitamin E

## Abstract

It is well-recognized that there is a need for medicine to migrate to a platform of delivering preventative care based on an individual's genetic make-up. The US National Research Council, the National Institute of Health and the American Heart Association all support the concept of utilizing genomic information to enhance the clinical management of patients. It is believed this type of precision healthcare will revolutionize health management. This current attitude of some of the most respected institutes in healthcare sets the stage for the utilization of the haptoglobin (Hp) genotype to guide precision management in type 2 diabetics (DM). There are three main Hp genotypes: 1-1, 2-1, 2-2. The Hp genotype has been studied extensively in (DM) and from the accumulated data it is clear that Hp should be considered in all DM patients as an additional independent cardiovascular disease (CVD) risk factor. In DM patients Hp2-2 generates five times increased risk of CVD compared to Hp1-1 and three times increased risk compared to Hp2-1. Data has also shown that carrying the Hp2-2 gene in DM compared to carrying an Hp1-1 genotype can increase the risk the microvascular complications of nephropathy and retinopathy. In addition, the Hp2-2 gene enhances post percutaneous coronary intervention (PCI) complications such as, in stent restenosis and need for additional revascularization during the first-year post PCI. Studies have demonstrated significant mitigation of CVD risk in Hp2-2 DM patients with administration of vitamin E and maintaining tight glycemic control. CVD is the leading cause of death and disability in DM as well-representing a huge financial burden. As such, evaluating the Hp genotype in DM patients can enhance the predictability and management of CVD risk.

## Introduction

The Hp genotype has been studied extensively in type II diabetes (DM). The current number of Americans diagnosed with DM is approximately 21 million (1). It was demonstrated years ago that DM patients carry the same risk for a heart attack as a non-DM patient who have already suffered a heart attack (2). Over 50% of all DM patients die from coronary heart disease (CHD). This knowledge provided the basis for aggressive management of cardiovascular (CV) risk factors in all DM patients (3). CVD represents the most deadly and costliest complication of DM (4). Treatment of CVD in DM patients is one of the most significant financial burdens on the healthcare system of the United States (5). Current evidence suggests that the Hp2-2 genotype in DM patients independently increases CV risk. A case- controlled study from a 6-year longitudinal population based study of DM patients examined 206 cases of CVD and 206 matched non-CVD cases for association of increased CV risk with Hp2-2 genotype. Multivariate analyses was performed adjusting for the following CV risk factors: HbA1c, fasting glucose and insulin, and family history of DM, low-density lipoprotein and high-density lipoprotein cholesterol, triglycerides, cigarette use, systolic blood pressure, body mass index, and family history of CVD. The results demonstrated that patients with Hp2-2 DM carried five times increased risk of CVD compared to Hp1-1 and three times increased risk of CVD compared to Hp2-1 DM patients with respective *p*-values of 0.002 and 0.010 (6). A more recent meta-analysis of studies confirmed the increased CVD risk in DM patient who are Hp2-2 compared to being non-Hp2-2. Three case controlled studies including the above study demonstrated a significant odds ratio of 2.2 for CV events; five cohort studies showed a significant odds ratio of 1.3 for CV events; three retrospective analysis of randomized controlled trials (RCT) indicated a significant odds ratio of 1.6 for CV events. The overall odds ratio for a CV event being an Hp2-2 DM compared to a non-Hp2-2 DM was a significant 1.44. These studies combined involved DM patients of which 6,161 were non-Hp2-2 and 4,684 were Hp2-2. They provide strong evidence that Hp2-2 genotype is a risk factor for CVD in DM (7). Data has also shown that carrying the Hp2-2 gene in DM compared to being an Hp1-1 DM increases the risk the microvascular complications of nephropathy and retinopathy. In addition, the Hp2-2 gene enhances post percutaneous coronary intervention (PCI) complications such as, in stent restenosis and need for additional revascularization during the first-year post PCI (8). Evaluating the Hp genotype in a DM patient can provide a more precise evaluation of the patient's CV risk.

Despite the above evidence, the associated increased CV risk of being an Hp2-2 diabetic is not without controversy. The large genome wide association study (GWAS) CARDIoGRAMplusC4D consortium called into question this association as none of the Hp single-nucleotide polymorphisms (SNPs) showed any increased risk of CAD in DM (9). However, Hp2 is a bi-allelic copy number variant (CNV). It is not a SNP and cannot be identified in the current SNP-Based GWA studies. Therefore, GWA studies cannot be used to assess the association of Hp2 with CV risk (10).

## Haptoglobin basic science

**Figure 1:**
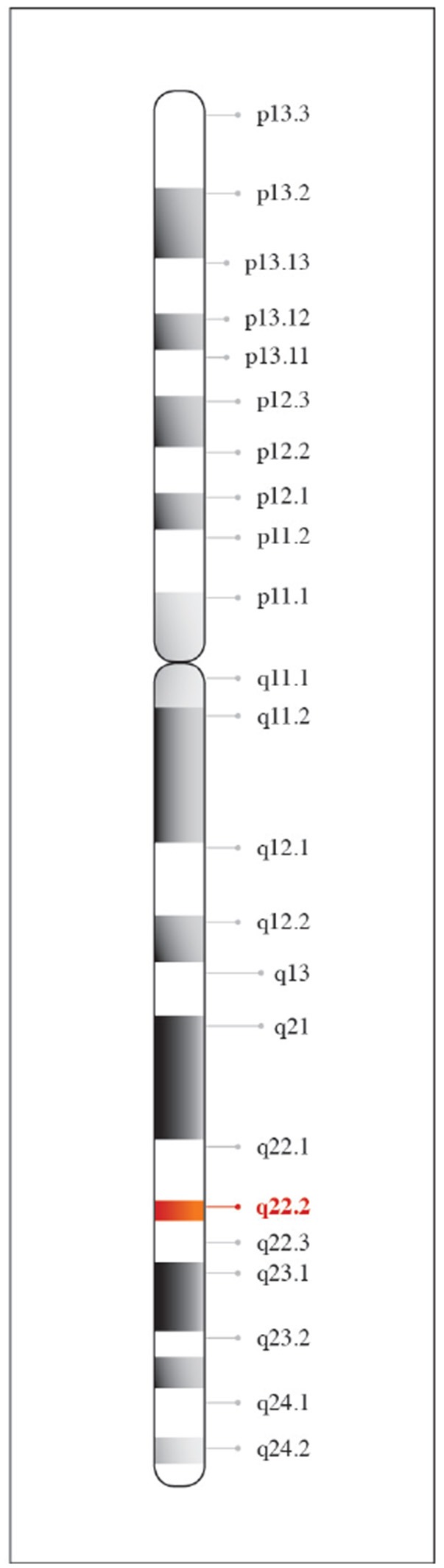
Chromosomal location of haptoglobin. Haptoglobin is found on chromosome 16 at position q22.2.

The biochemical and physiologic properties of Hp were described more than 75 years ago (11–13). The chromosomal location for the human Hp gene is 16q22.2 (Figure 1) and gene products are represented by three structural alleles; Hp1F, Hp1S, and Hp2 (14). Structural composition of Hp1F and Hp1S differs by one amino acid at position 54 (lysine vs. glutamic acid) (15), the Hp2 allele results from a fusion of the Hp1F and Hp1S structural alleles.

Haptoglobin is an acute phase α_2_-glycoprotein produced in the liver with a major function in the physiological recycling of free hemoglobin (Hb). Biologically, Hp has high affinity to Hb in order to promote iron conservation following hemolysis (16, 17) and to eliminate the risk of hemoglobin-mediated renal injury (18–20). As a clearance protein, Hp has a principal function in the elimination of free iron oxidative potential.

**Figure 2:**
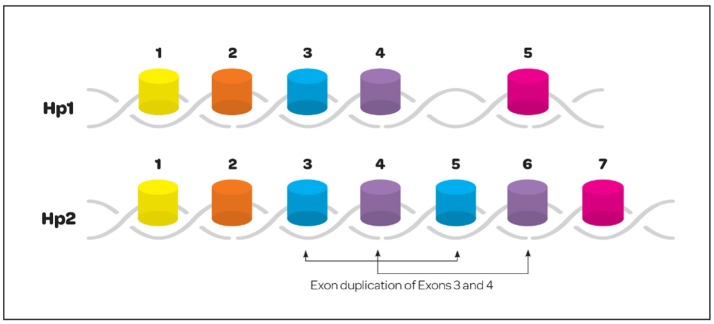
Exonic structure of the haptoglobin 1 and 2 alleles. Hp polymorphism is only seen in the human. Polymorphism resulted from a crossover gene duplication event involving exons 3 and 4. The resulting gene structure creates a 5 exon Hp1 and 7 exon Hp2.

**Figure 3:**
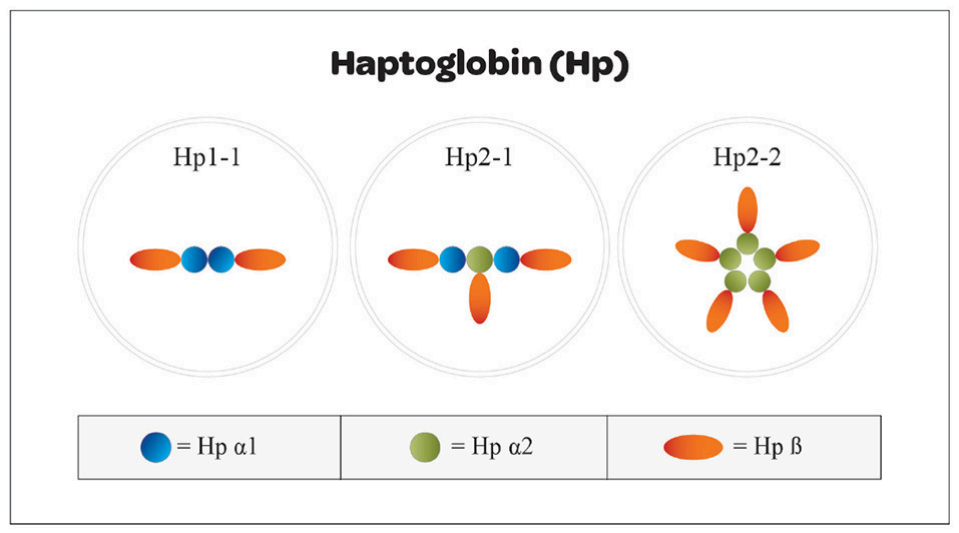
Structural arrangement of Hp1 and Hp2 oligomers. Haptoglobin can be found in a variety of structural conformations resulting from combinations of Hp1 and Hp2 subunits that mediate the accessibility to the tissue spaces. Oligomers that are too large cannot enter into the tissue space and effect neutralization of free heme molecules. Hp1 homodimers have greater binding efficiency and easier access than do Hp2-1 heterodimers or Hp2-2 oligomers.

Haptoglobin is found in all mammalian serum, however, polymorphism has only been noted in humans (21). It is generally believed that polymorphism was an adaptive advantage in years past against pathogens. The polymorphism seen in humans arose from a crossover duplication of exons 3 and 4; that has resulted in an Hp1 molecule with 5 exons and an Hp2 molecule containing 7 exons (Figure 2) (22). Three multimeric genotypes have been described and identified by gel electrophoresis as Hp1-1, Hp2-1, and Hp2-2 (23, 24). Hp1 and Hp2 alleles in humans result in the production of several structural multimers that include Hp1-1 dimers, Hp2-1 heterodimers and Hp2-2 dimers (Figure 3). The general frequency of each genotype in the United States is 15–18% Hp1-1, 46% Hp2-1 and 36% Hp2-2 (25), however, the frequency of Hp2-2 among various ethnic and racial groups has been found to be significantly higher than 36% (26).

Haptoglobin has a principal function in the clearance of hemoglobin (Hb) from the vasculature and intravascular spaces. Free Hb is routinely released into tissues as result of intravascular hemolysis following the destruction and recycling of senescent red blood cells (RBC), this process occurs at a rate of 2 × 10^6^ RBC per second (27, 28). Severe complications result from intravascular hemolysis when accompanied by pathologies such as cancer, infectious disease, trauma, and diabetes (29, 30). Haptoglobin binds to Hb to form strong non-covalent complexes that facilitate removal via CD163 receptor-mediated endocytosis on hepatocytes, Kupffer cells and tissue macrophages through the reticuloendothelial system (31–34). Concentrations of Hp within the body are sufficient to bind and clear 3 grams of Hb (38–208 mg/dL), effectively eliminating free Hb from circulation (35). The binding of Hp to free Hb represents one of the highest protein-protein affinities found in nature (17, 36, 37). This high affinity observation highlights the importance of this biological process to the protection of the organism from oxidative damage. Significant deleterious consequences arise within tissues when free iron remains in circulation. It is well-established in the literature that iron overload contributes to the exacerbation and development of CVD and DM (38–41). The pathophysiology of iron derives from the generation of hydroxyl radical and other reactive oxygen species (42). The ability of Hp to reduce the tissue damaging effects from free radicals is genotype dependent and critical to organismal homeostasis. Molecular differences in Hp structures act as mechanism that can exclude the entry of Hp molecules into the intravascular spaces where the molecules can engage and neutralize the effect of free iron; as a result, in individuals with the Hp2-1 or Hp2-2 genotypes Hb remains in the circulation for extended periods of time resulting in heightened oxidative stress to the vasculature (43). The loss of antioxidant capacity resulting from Hp polymorphism in the diabetic patient is important to clinically assess. In particular, the reduced antioxidant capacity of the Hp2-2 genotype has been shown to be a susceptibility marker for DM patients that contributes to complications (44).

## Clinical perspective of haptoglobin polymorphism

Including Hp testing into the clinical arena allows for precision based healthcare decisions on the CVD vulnerability of the DM patient. These patients have complex CV health issues that require individualized attention. High-density lipoprotein (HDL) particle is a modifiable CV risk in the management of patients with DM. The levels of HDL are generally low in DM and functionality diminished (45). Patients with DM and Hp2-2 genotype are at increased risk for the Hp-Hb complex attaching to HDL particles. Hp2-2 has an altered binding affinity for CD163 on monocytes, which is 8 times less than the affinity for Hp1-1. In addition, Hp2-2 DM patients have about half the amount of CD163 ligands on monocytes as compared to Hp2-2 non-DM patients. These two factors result in greater amounts of the Hp-Hb complex in the serum with the ability to bind to HDL. Binding of Hp-Hb complex to HDL particles results in oxidative stress and dysfunctional HDL particles. It has been demonstrated that treating Hp2-2 DM patients with 400IU natural source d-alpha tocopherol (Vitamin E) for 2 months significantly improved HDL particle efflux capacity (46). Demonstrated in a separate trial, treatment with supplemental 400IU of natural source d-alpha tocopherol for 3 months improved HDL particle functionality in Hp2-2 DM patients, but decreased functionality in Hp2-1 DM patients (7, 47). These findings may help explain evidence from two studies indicating a substantial reduction in CV risk with the use of supplemental vitamin E in Hp2-2 DM, but no benefit in Hp1-1 or Hp2-1 DM patients (48, 49). A randomized, placebo controlled, double-blinded trial of 2,967 DM patients with 48% being Hp2-2 was performed. Half of the Hp2-2 DM patients were randomized to 400IU of natural source d-alpha tocopherol and the other patients received placebo. The trial was stopped after 18 months due to the significant drop in CV risk in the DM Hp2-2 patients receiving vitamin E. The intent to treat arm, significantly demonstrated a reduced risk by over 200% and in the patients taking the vitamin, the risk was reduced almost 300% (50). When this study was recently included in a meta-analysis with two other RCTs of DM Hp2-2 patients, the number of patients receiving vitamin E therapy was 1,094 and 1.016 receiving placebo. The dose of vitamin E was 400 IU a day in 948 of the patients and 600 IU every other day in 146 patients. Those treated with vitamin E had a significant 34% reduction in incidence of CV events. In two of those RCTs 541 non-Hp2-2 DM patients received therapy with vitamin E and 582 who received placebo. The dose of vitamin E was 400 IU in 326 patients and 600 IU every other day in 215 patients. Those treated had a non-significant 11% increased risk of CV events. These studies indicate vitamin E may be a cost effective therapy to reduce CV risk in DM (7). In alliance with these studies, it was recently demonstrated that vitamin E is decreased in the HDL particle of Hp2-2 individuals (51).

Managing CVD in DM patients drives around $200 billion of annual healthcare costs in USA. Vitamin E appears to provide significant benefit for the high CV risk in Hp2-2 DM patients (7). This therapy is widely available and inexpensive. Embracing this opportunity for precision based health care decisions, makes sense to reduce the mortality, morbidity, and financial burden of DM (52).

The latest guidelines for glycemic control issued in March of 2018 call for a softening of glucose control (53). The new guidelines state that achieving an HbA1c goal in most DM patients of between 7 and 8% should be the target and the intensity of glycemic intervention be reduced in DM patients with an HbA1c < 6.5% (54). The evidence indicates this may be detrimental advice for Hp2-2 DM patients. A nested case-control study from the Health Professional Follow-up Study (HPFS) involving 695 heart attack patients and 696 non-heart attack patients during 16-years of follow-up was evaluated for increased risk due to Hp2-2 genotype in DM and HbA1c values >6.5% vs. < 6.5%. Risk was adjusted for alcohol intake, diet, body mass index (BMI), hypertension, hypercholesterolemia, family history of CHD and medications. After this multivariate adjustment the relative risk for heart attack was 3.07 times greater for Hp2-2 DM with a HbA1c >6.5% (55). A similar nested case control study was performed with data from the Nurses' Health Study (NHS), which involved 404 heart attack patients and 400 controls during a 14-year follow-up (55). The relative risk for a heart attack in Hp2-2 DM patients with an HbA1c >6.5% was 10.59 times greater. This relative risk was found to be significant and dramatic during the first 8 years of the 14–16-year follow-up with a relative risk over 28 times higher. During the remaining 6–8 years of follow-up, there was an insignificant 5 times increased risk. When Hp2-2 DM patients with HbA1c < 6.5% were compared to non-Hp2-2 DM patients with a HbA1c < 6.5%, there was no significant increased risk in the Hp2-2 patients. No relationship to glycemic control and heart attack risk was demonstrated in non-Hp2-2 DM patients. These results should be tested in a randomized trial.

Mechanistically, the relationship between Hp2-2 status and glycemic control is believed to involve heightened oxidative stress. Glycosylation of Hb is an additional factor that reduces the effectiveness of Hp2-2 to perform as an antioxidant. Therefore, when the Hp-Hb complex attaches to HDL particles it generates oxidation of HDL-particles and its components, such as glutathione and apolipoprotein A. This modification creates an HDL particle, which is proatherogenic and prothrombotic. Obtaining the Hp genotype of the DM patient would allow for precision glycemic management. This is a clear example of entering an age where we can render care to an individual patient based on their biological uniqueness. Guidelines treat patients as averages of the study populations. Personalized and precision care through genotyping should promote better outcomes. Hp genotyping would assist in establishing glycemic control goals in DM patients (55).

## Arterial damage and cardiovascular risk

Hp2-2 is structurally too large to enter the intimal space of the artery wall. This can be particularly detrimental in a patient with DM who presents with active arterial disease. Progression of atherosclerosis in DM has been demonstrated to involve neovascularization and intra-plaque hemorrhage, leading to the further release of Hb (56). Because the Hp2-2 oligomer cannot enter the arterial wall to bind and neutralize the Hb, oxidative stress increases. Evidence demonstrates that since Hp2-2 cannot penetrate into the intimal space, increased levels of myeloperoxidase ensues, which can destabilize plaque and result in an atherothrombotic event (57). Identifying a DM patient with Hp2-2 genotype can alert the clinician of the critical importance of assessing and optimally managing the myriad of conditions that inflame the artery and drive an active atherosclerotic disease process. It has been demonstrated that a clinically based comprehensive CVD risk reduction program can halt atherosclerosis and de-lipidate arterial plaque (58). Three prospective studies have now demonstrated a direct relationship between lipid richness in a plaque and risk of a CV event (59–61). Discovering that the DM patient is Hp2-2 identifies a patient in need of comprehensive and precision-based CV risk factor management.

It is becoming clearer that Hp2-2 genotype is a risk factor for the development of atherosclerosis, aneurysm, and carotid stenosis that is independent of other classical risk factors such as smoking, hypertension, high cholesterol, diabetes, and hyperhomocystenemia (20, 62, 63). Lioupis et al. demonstrated higher concentrations of iron in the atherosclerotic plaque of male diabetic patients with the Hp2-2 genotype (50). The suggestion from this work is that increased intraplaque iron may be responsible for an elevation in oxidative stress and further destabilization of the plaque (64). As pertains to the development of refractory hypertension, Hp2-2 genotype is an additional risk factor to be considered in patients with existing hypertension (65, 66). Patients identified with the Hp2-2 genotype require additional antihypertensive therapy when compared to patients of other genotypes. Hp2-2 patients require additional, directed medical support, and follow-up than do patients with other Hp genotypes (65, 66). The rate of complication in patients with Hp1-1 is lower than the rate for hypertensive patients with other Hp genotypes (66).

The hypothesis that patients with Hp2-2 genotype are far more vulnerable to oxidative damage in arteries with existing atherosclerotic plaques is supported by the literature and testing patients for Hp2-2 genotype will be of clinical merit and it's utility is likewise supported by the literature. Data and literature accumulated over the past two decades clearly illustrates that Hp genotyping should be considered as a predictor of cardiovascular complication and a measure of future patient prognosis and outcome.

## Best practices and therapeutic intervention

As noted by Cheng et al. personalized medicine involves the “coupling of established clinical and pathological indexes with state-of-the-art molecular profiling to create diagnostic, prognostic, and therapeutic strategies precisely tailored to each patient's requirements—hence the term precision medicine” (67). Treating patients as unique individuals allows for optimal care and improved outcomes (68). Appreciating our most vulnerable population in the CVD community, the DM individuals, have a >50% risk of dying from CHD. The current number of Americans diagnosed with DM is approximately 21 million. Incorporating Hp genotype testing with this group of patients, allows for a more precise and calculated assessment and treatment plan.

In terms of assessing for CV risk, it now appreciated that identifying subclinical coronary, carotid or femoral atherosclerosis has bearing on risk for heart attack, stroke and CV death. Discovering such disease can enhance patient management and increase compliance (69, 70) Such testing can better define the presence, extent and location of atherosclerosis which has more bearing on CV risk than simply the stenosis of disease (71). Due to the substantially heightened risk of CV events in Hp2-2 DM patients, it would seem reasonable to do imaging for subclinical disease. Finding such disease would confirm their high risk and enhance management decisions.

Inflammation is the cause of arterial disease and the trigger for CV events (72, 73). Therefore, it would be reasonable to measure biomarkers of arterial inflammation in Hp2-2 DM patients. The markers of endothelial inflammation and dysfunction, namely, hsCRP, fibrinogen and microalbumin-creatinine ratio, could be assessed (74, 75). Lipoprotein-associated phospholipase A2 activity should be measured at baseline to assess intimal atherosclerotic activity (76). This biomarker could then be monitored for changes to indicate CV event risk (77). Urine F2 isoprostane could be measured to evaluate the degree of oxidative stress and patient compliance with lifestyle issues (78). These biomarkers of inflammation would indicate the patient's current risk for a CV event and potential need for additional therapies.

In terms of therapy, the current guidelines for DM do an excellent job addressing blood pressure goals and lipid goals however, the event rates and recidivistic rates of CVD in DM continue to create substantial financial and personal loss (53). It was reported from a prospective analysis of 8,970 women and 2,557 men recently diagnosed with DM that compliance with lifestyle can significantly reduce CVD risk. These patients were followed just over 13 years. A multivariate adjustment for the following CV risk factors was performed: age, sex, ethnicity, body mass index at diabetes diagnosis, menopausal status, family history of diabetes, family history of myocardial infarction, current aspirin use, current multivitamin use, and diabetes duration. Four lifestyle elements were assessed: alcohol, diet, physical activity, and smoking. The analysis demonstrated for each lifestyle element in place there was a 12% lower risk of CHD, a 21% lower risk of stroke and a 27% lower risk of CVD death (79). This information would need to be convincingly conveyed to all Hp2-2 DM.

In addition to the above strategies to mitigate CVD risk, clinicians should strive to optimally manage other known CV risk factors in the high risk Hp2-2 DM population. These factors include: sleep, psychosocial issues, periodontal disease, endodontic disease, and other inflammatory diseases (80–84). Applying this type of comprehensive management in a real clinical practice has been shown to have a positive effect on the atherosclerotic disease process (68, 85). It has been proposed that a paradigm shift needs to occur in healthcare placing more emphasis on prevention management which will generate more value for patients and most likely substantial cost effectiveness (86). The high risk Hp2-2 DM population is a prime target for such preventive care. Applying haptoglobin testing to all DM patients creates an opportunity for precision based clinical assessment and therapeutic decisions. This information provides the clinician the precision opportunity to target this population for intensive prevention services. These items could include emerging technologies for imaging subclinical disease and biomarkers of arterial inflammation. These patients deserve to be educated on the importance of managing traditional known CV risk factors and the importance of compliance with lifestyle. They should also be educated on other emerging risk factors that can generate arterial inflammation. Assessment for and management of any of these risk factors should be undertaken. In addition, given our current scientific knowledge, Hp2-2 DM patients should be treated with a cost-effective treatment of 400IU natural source d-alpha tocopherol. Such therapy has been shown to enhance the ability to stabilize the atherogenic disease process in this vulnerable group. This treatment goes beyond the guidelines and allows for individualized therapeutic interventions. Also, in light of our current evidence, these patients should strive to maintain glycemic control with an HbA1c < 6.5%, which reaches beyond the current overall guidelines for DM.

A randomized prospective study should be done in Hp2-2 DM patients to assess the CV risk reduction achieved with the above proposed comprehensive assessment and management vs. the current standard of care for DM patients. Another study should be done to determine if there is any potential benefit for vitamin E therapy in Hp2-2 individuals along the continuum of insulin resistance prior to the diagnosis of DM. In the interim, since Hp testing is arguably inexpensive with a cost of >$300 up front and obviously inexpensive long term as it is a one-time test, it seems reasonable with the current scientific knowledge to measure Hp genotype in all DM patients. Hp testing augments the current standards in this vulnerable population yielding the opportunity to dramatically improve patient outcomes. Approximately, one third (~ 7 million) will be Hp2-2. Those individuals can be targeted for additional assessment and management beyond current guidelines. Such care may help stimulate our current system to a more value based prevention model. Hp testing for DM patients is in direct support of the American Heart Association's mission to practice precision healthcare, especially in the most vulnerable CV patient populations, such as the DM patient. Hp testing in DM has the opportunity to enhance personalized and precision healthcare.

## Author contributions

The authors contributed equally to the writing and development of the manuscript.

### Conflict of interest statement

ALD and BFB are Medical Consultants for Mygenetx Clinical Laboratory. The remaining author declares that the research was conducted in the absence of any commercial or financial relationships that could be construed as a potential conflict of interest.

## References

[1] Alam SR Shah AS Richards J Lang NN Barnes G Joshi N. Ultrasmall superparamagnetic particles of iron oxide in patients with acute myocardial infarction: early clinical experience. Circ Cardiovasc Imag. (2012) 5:559–65. 10.1161/CIRCIMAGING.112.974907 22875883

[2] Alayash AI Andersen CB Moestrup SK Bulow L. Haptoglobin: the hemoglobin detoxifier in plasma. Trends Biotechnol. (2013) 31:2–3. 10.1016/j.tibtech.2012.10.003 23140673

[3] American Diabetes Association Economic costs of diabetes in the U.S. in 2012. Diabetes Care (2013) 36:1033–46. 10.2337/dc12-2625 23468086 PMC3609540

[4] American Diabetes Association 6. Glycemic targets: standards of medical care in diabetes-2018. Diabetes Care (2018) 41:S55–64. 10.2337/dc18-S006 29222377

[5] Amiri AA Hashemi-Soteh MB Haghshenas MR Daneshvar F Rastegar A Farazmand T. Haptoglobin polymorphism in individuals with type 2 diabetic microangiopathy. N Am J Med Sci. (2013) 5:529–35. 10.4103/1947-2714.118929 24251270 PMC3818825

[6] Arbab-Zadeh A Fuster V. The risk continuum of atherosclerosis and its implications for defining CHD by coronary angiography. J Am Coll Cardiol. (2016) 68:2467–78. 10.1016/j.jacc.2016.08.069 27908353

[7] Ascenzi P Bocedi A Visca P Altruda F Tolosano E Beringhelli T. Hemoglobin and heme scavenging. IUBMB Life (2005) 57:749–59. 10.1080/15216540500380871 16511968

[8] Asleh R Blum S Kalet-Litman S Alshiek J Miller-Lotan R Asaf R. Correction of HDL dysfunction in individuals with diabetes and the haptoglobin 2-2 genotype. Diabetes (2008) 57:2794–800. 10.2337/db08-0450 18599520 PMC2551691

[9] Asleh R Briasoulis A Berinstein EM Wiener JB Palla M Kushwaha SS. Meta-analysis of the association of the haptoglobin genotype with cardiovascular outcomes and the pharmacogenomic interactions with vitamin E supplementation. Pharmgenomics Pers Med. (2018) 11:71–82. 10.2147/PGPM.S159454 29731659 PMC5923226

[10] Baber U Mehran R Sartori S Schoos MM Sillesen H Muntendam P. Prevalence, impact, and predictive value of detecting subclinical coronary and carotid atherosclerosis in asymptomatic adults: the BioImage study. J Am Coll Cardiol. (2015) 65:1065–74. 10.1016/j.jacc.2015.01.017 25790876

[11] Baek JH D'agnillo F Vallelian F Pereira CP Williams MC Jia Y. Hemoglobin-driven pathophysiology is an *in vivo* consequence of the red blood cell storage lesion that can be attenuated in guinea pigs by haptoglobin therapy. J Clin Invest. (2012) 122:1444–58. 10.1172/JCI59770 22446185 PMC3314461

[12] Bale B Doneen A Cool LC Beat the Heart Attack Gene: The Revolutionary Plan to Prevent Heart Disease, Stroke, and Diabetes New York, NY: Wiley (2014).

[13] Bale BF Doneen AL Vigerust DJ. High-risk periodontal pathogens contribute to the pathogenesis of atherosclerosis. Postgrad Med J. (2017) 93:215–20. 10.1136/postgradmedj-2016-134279 27899684 PMC5520251

[14] Bensi G Raugei G Klefenz H Cortese R. Structure and expression of the human haptoglobin locus. EMBO J. (1985) 4:119–26. 10.1002/j.1460-2075.1985.tb02325.x 4018023 PMC554159

[15] Blum S Vardi M Brown JB Russell A Milman U Shapira C. Vitamin E reduces cardiovascular disease in individuals with diabetes mellitus and the haptoglobin 2-2 genotype. Pharmacogenomics (2010) 11:675–84. 10.2217/pgs.10.17 20415560 PMC2880717

[16] Bowman BH Barnett DR Lum JB Yang F. Haptoglobin. Methods Enzymol. (1988) 163:452–74. 10.1016/0076-6879(88)63043-6 3148829

[17] Bowman BH Kurosky A. Haptoglobin: the evolutionary product of duplication, unequal crossing over, and point mutation. Adv Hum Genet. (1982) 12:189–261, 453–184. 6751044 10.1007/978-1-4615-8315-8_3

[18] Bullard KM Cowie CC Lessem SE Saydah SH Menke A Geiss LS. Prevalence of diagnosed diabetes in adults by diabetes type - United States, 2016. MMWR Morb Mortal Wkly Rep. (2018) 67:359–61. 10.15585/mmwr.mm6712a2 29596402 PMC5877361

[19] Cahill LE Jensen MK Chasman DI Hazra A Levy AP Rimm EB. Currently available versions of genome-wide association studies cannot be used to query the common haptoglobin copy number variant. J Am Coll Cardiol. (2013) 62:860–1. 10.1016/j.jacc.2013.04.079 23747761 PMC3919047

[20] Cahill LE Jensen MK Chiuve SE Shalom H Pai JK Flint AJ. The risk of coronary heart disease associated with glycosylated hemoglobin of 6.5% or greater is pronounced in the Haptoglobin 2-2 Genotype. J Am Coll Cardiol. (2015) 66:1791–99. 10.1016/j.jacc.2015.07.076 26483103 PMC4616252

[21] Carter K Worwood M. Haptoglobin: a review of the major allele frequencies worldwide and their association with diseases. Int J Lab Hematol. (2007) 29:92–110. 10.1111/j.1751-553X.2007.00898.x 17474882

[22] Cheng HG Patel BS Martin SS Blaha M Doneen A Bale B. Effect of comprehensive cardiovascular disease risk management on longitudinal changes in carotid artery intima-media thickness in a community-based prevention clinic. Arch Med Sci. (2016) 12:728–35. 10.5114/aoms.2016.60955 27478452 PMC4947619

[23] CARDIoGRAMplusC4D Consortium Deloukas P Kanoni S Willenborg C Farrall M Assimes TL Large-scale association analysis identifies new risk loci for coronary artery disease. Nat Genet. (2013) 45:25–33. 10.1038/ng.2480 23202125 PMC3679547

[24] Delanghe JR Duprez DA De Buyzere ML Bergez BM Callens BY Leroux-Roels GG. Haptoglobin polymorphism and complications in established essential arterial hypertension. J Hypertens. (1993) 11:861–7. 10.1097/00004872-199308000-00013 8228210

[25] Delanghe JR Duprez DA De Buyzere ML Bergez BM Claeys LR Leroux-Roels GG. Refractory hypertension is associated with the haptoglobin 2-2 phenotype. J Cardiovasc Risk (1995) 2:131–6. 10.1097/00043798-199504000-00008 7606649

[26] Dregan A Charlton J Chowienczyk P Gulliford MC. Chronic inflammatory disorders and risk of type 2 diabetes mellitus, coronary heart disease, and stroke: a population-based cohort study. Circulation (2014) 130:837–44. 10.1161/CIRCULATIONAHA.114.009990 24970784

[27] Durnford A Dunbar J Galea J Bulters D Nicoll JA Boche D. Haemoglobin scavenging after subarachnoid haemorrhage. Acta Neurochir Suppl. (2015) 120:51–4. 10.1007/978-3-319-04981-6_9 25366599

[28] Emerging Risk Factors Collaboration Kaptoge S Di Angelantonio E Pennells L Wood AM White IR. C-reactive protein, fibrinogen, and cardiovascular disease prediction. N Engl J Med. (2012) 367:1310–20. 10.1056/NEJMoa1107477 23034020 PMC3714101

[29] Farbstein D Blum S Pollak M Asaf R Viener HL Lache O. Vitamin E therapy results in a reduction in HDL function in individuals with diabetes and the haptoglobin 2-1 genotype. Atherosclerosis (2011) 219:240–4. 10.1016/j.atherosclerosis.2011.06.005 21722898 PMC3200506

[30] Feng D Esperat MC Doneen AL Bale B Song H Green AE. Eight-year outcomes of a program for early prevention of cardiovascular events: a growth-curve analysis. J Cardiovasc Nurs. (2015) 30:281–91. 10.1097/JCN.0000000000000141 24717191

[31] Ferguson JF Hinkle CC Mehta NN Bagheri R Derohannessian SL Shah R Translational studies of lipoprotein-associated phospholipase A(2) in inflammation and atherosclerosis. J Am Coll Cardiol. (2012) 59:764–72. 10.1016/j.jacc.2011.11.019 22340269 PMC3285416

[32] Goldenstein H Levy NS Levy AP. Haptoglobin genotype and its role in determining heme-iron mediated vascular disease. Pharmacol Res. (2012) 66:1–6. 10.1016/j.phrs.2012.02.011 22465143 PMC3345090

[33] Goldenstein H Levy NS Ward J Costacou T Levy AP. Haptoglobin genotype is a determinant of hemoglobin adducts and Vitamin E content in HDL. J Diabetes Res. (2018) 2018:6125420. 29888289 10.1155/2018/6125420 PMC5985109

[34] Haffner SM Lehto S Ronnemaa T Pyorala K Laakso M. Mortality from coronary heart disease in subjects with type 2 diabetes and in nondiabetic subjects with and without prior myocardial infarction. N Engl J Med. (1998) 339:229–34. 10.1056/NEJM199807233390404 9673301

[35] Halliwell B. Free radicals and antioxidants: updating a personal view. Nutr Rev. (2012) 70:257–65. 10.1111/j.1753-4887.2012.00476.x 22537212

[36] Hansson GK. Inflammation and Atherosclerosis: the end of a controversy. Circulation (2017) 136:1875–7. 10.1161/CIRCULATIONAHA.117.030484 28916641

[37] Heart Outcomes Prevention Evaluation Study Investigators Yusuf S Dagenais G Pogue J Bosch J Sleight P. Vitamin E supplementation and cardiovascular events in high-risk patients. N Engl J Med. (2000) 342:154–60. 10.1056/NEJM200001203420302 10639540

[38] Howard BV Magee MF. Diabetes and cardiovascular disease. Curr Atheroscler Rep. (2000) 2:476–81. 10.1007/s11883-000-0046-8 11122781

[39] Ijas P Saksi J Soinne L Tuimala J Jauhiainen M Jula A. Haptoglobin 2 allele associates with unstable carotid plaque and major cardiovascular events. Atherosclerosis (2013) 230:228–34. 10.1016/j.atherosclerosis.2013.07.008 24075749

[40] Irwin DC Hyen Baek J Hassell K Nuss R Eigenberger P Lisk C. Hemoglobin-induced lung vascular oxidation, inflammation, and remodeling contribute to the progression of hypoxic pulmonary hypertension and is attenuated in rats with repeated-dose haptoglobin administration. Free Radic Biol Med. (2015) 82:50–62. 10.1016/j.freeradbiomed.2015.01.012 25656991 PMC4387123

[41] Javaheri S Barbe F Campos-Rodriguez F Dempsey JA Khayat R Javaheri S. Sleep apnea: types, mechanisms, and clinical cardiovascular consequences. J Am Coll Cardiol. (2017) 69:841–58. 10.1016/j.jacc.2016.11.069 28209226 PMC5393905

[42] Jayle MF Said I Gillard P Action of haptoglobin on peroxidase catalysis of hemoglobin: new theory on the formation of enzymes. Bull Soc Chim Biol. (1946) 28:63–80. 20988267

[43] Juutilainen A Lehto S Ronnemaa T Pyorala K Laakso M Type 2 diabetes as a “coro nary heart disease equivalent”: an 18-year prospective population-based study in Finnish subjects. Diabetes Care (2005) 28:2901–7. 10.2337/diacare.28.12.2901 16306552

[44] Kaempfer T Duerst E Gehrig P Roschitzki B Rutishauser D Grossmann J. Extracellular hemoglobin polarizes the macrophage proteome toward Hb-clearance, enhanced antioxidant capacity and suppressed HLA class 2 expression. J Proteome Res. (2011) 10:2397–408. 10.1021/pr101230y 21405025

[45] Kato GJ. Haptoglobin halts hemoglobin's havoc. J Clin Invest. (2009) 119:2140–2. 10.1172/JCI40258 19620777 PMC2719939

[46] Kristiansen M Graversen JH Jacobsen C Sonne O Hoffman HJ Law SK. Identification of the haemoglobin scavenger receptor. Nature (2001) 409:198–201. 10.1038/35051594 11196644

[47] Langlois MR Delanghe JR. Biological and clinical significance of haptoglobin polymorphism in humans. Clin Chem. (1996) 42:1589–600. 8855140

[48] Levy AP Hochberg I Jablonski K Resnick HE Lee ET Best L. Haptoglobin phenotype is an independent risk factor for cardiovascular disease in individuals with diabetes: the strong heart study. J Am Coll Cardiol. (2002) 40:1984–90. 10.1016/S0735-1097(02)02534-2 12475459

[49] Lim SK. Consequences of haemolysis without haptoglobin. Redox Rep. (2001) 6:375–8. 10.1179/135100001101536571 11865980

[50] Lioupis C Barbatis C Drougou A Koliaraki V Mamalaki A Klonaris C. Association of haptoglobin genotype and common cardiovascular risk factors with the amount of iron in atherosclerotic carotid plaques. Atherosclerosis (2011) 216:131–8. 10.1016/j.atherosclerosis.2011.01.028 21316675

[51] Lipiski M Deuel JW Baek JH Engelsberger WR Buehler PW Schaer DJ. Human Hp1-1 and Hp2-2 phenotype-specific haptoglobin therapeutics are both effective in vitro and in guinea pigs to attenuate hemoglobin toxicity. Antioxid Redox Signal (2013) 19:1619–33. 10.1089/ars.2012.5089 23418677 PMC3809386

[52] Liu G Li Y Hu Y Zong G Li S Rimm EB. Influence of lifestyle on incident cardiovascular disease and mortality in patients with diabetes mellitus. J Am Coll Cardiol. (2018) 71:2867–76. 10.1016/j.jacc.2018.04.027 29929608 PMC6052788

[53] Madder RD Husaini M Davis AT Vanoosterhout S Khan M Wohns D. Large lipid-rich coronary plaques detected by near-infrared spectroscopy at non-stented sites in the target artery identify patients likely to experience future major adverse cardiovascular events. Eur Heart J Cardiovasc Imag. (2016) 17:393–9. 10.1093/ehjci/jev340 26800770

[54] Mccormick DJ Atassi MZ. Hemoglobin binding with haptoglobin: delineation of the haptoglobin binding site on the alpha-chain of human hemoglobin. J Protein Chem. (1990) 9:735–42. 10.1007/BF01024768 2073325

[55] Melamed-Frank M Lache O Enav BI Szafranek T Levy NS Ricklis RM. Structure-function analysis of the antioxidant properties of haptoglobin. Blood (2001) 98:3693–8. 10.1182/blood.V98.13.3693 11739174

[56] Milman U Blum S Shapira C Aronson D Miller-Lotan R Anbinder Y. Vitamin E supplementation reduces cardiovascular events in a subgroup of middle-aged individuals with both type 2 diabetes mellitus and the haptoglobin 2-2 genotype: a prospective double-blinded clinical trial. Arterioscler Thromb Vasc Biol. (2008) 28:341–7. 10.1161/ATVBAHA.107.153965 18032779

[57] Mirnezami R Nicholson J Darzi A. Preparing for precision medicine. N Engl J Med. (2012) 366:489–91. 10.1056/NEJMp1114866 22256780

[58] Morrow JD. Quantification of isoprostanes as indices of oxidant stress and the risk of atherosclerosis in humans. Arterioscler Thromb Vasc Biol. (2005) 25:279–86. 10.1161/01.ATV.0000152605.64964.c0 15591226

[59] Nantasenamat C Prachayasittikul V Bulow L. Molecular modeling of the human hemoglobin-haptoglobin complex sheds light on the protective mechanisms of haptoglobin. PLoS ONE (2013) 8:e62996. 10.1371/journal.pone.0062996 23638175 PMC3637213

[60] Nicolaides A Panayiotou AG. Screening for Atherosclerotic cardiovascular risk using ultrasound. J Am Coll Cardiol. (2016) 67:1275–7. 10.1016/j.jacc.2016.01.016 26988946

[61] Pascual JM Rodilla E Costa JA Garcia-Escrich M Gonzalez C Redon J. Prognostic value of microalbuminuria during antihypertensive treatment in essential hypertension. Hypertension (2014) 64:1228–34. 10.1161/HYPERTENSIONAHA.114.04273 25245391

[62] Pessi T Karhunen V Karjalainen PP Ylitalo A Airaksinen JK Niemi M. Bacterial signatures in thrombus aspirates of patients with myocardial infarction. Circulation (2013) 127:1219–28, e1211–6. 10.1161/CIRCULATIONAHA.112.001254 23418311

[63] Polonouski M. Biochemistry of haptoglobin and its clinical interpretation. Rend Ist Sup Sanit. (1950) 13:842–75. 14844800

[64] Polonovski M Influence of plasma globin on the index haptoglobin.Sang (1945) 16:496–8. 21014443

[65] Pryor K Volpp K. Deployment of preventive interventions - time for a paradigm shift. N Engl J Med. (2018) 378:1761–3. 10.1056/NEJMp1716272 29742382

[66] Purushothaman KR Meerarani P Moreno PR. Inflammation and neovascularization in diabetic atherosclerosis. Indian J Exp Biol. (2007) 45:93–102. Available online at: https://pdfs.semanticscholar.org/efcf/f30a7b47c56de96dd47992e8a2e67aed141f.pdf 17249333

[67] Purushothaman M Krishnan P Purushothaman KR Baber U Tarricone A Perez JS. Genotype-dependent impairment of hemoglobin clearance increases oxidative and inflammatory response in human diabetic atherosclerosis. Arterioscler Thromb Vasc Biol. (2012) 32:2769–75. 10.1161/ATVBAHA.112.252122 22982461

[68] Qaseem A Wilt TJ Kansagara D Horwitch C Barry MJ Forciea MA. Hemoglobin A1c targets for glycemic control with pharmacologic therapy for nonpregnant adults with type 2 diabetes mellitus: a guidance statement update from the American College of Physicians. Ann Intern Med. (2018) 168:569–76. 10.7326/M17-0939 29507945

[69] Rajapurkar MM Hegde U Bhattacharya A Alam MG Shah SV. Effect of deferiprone, an oral iron chelator, in diabetic and non-diabetic glomerular disease. Toxicol Mech Methods (2013) 23:5–10. 10.3109/15376516.2012.730558 22978744

[70] Ratanasopa K Chakane S Ilyas M Nantasenamat C Bulow L. Trapping of human hemoglobin by haptoglobin: molecular mechanisms and clinical applications. Antioxid Redox Signal (2013) 18:2364–74. 10.1089/ars.2012.4878 22900934

[71] Ridker PM Everett BM Thuren T Macfadyen JG Chang WH Ballantyne C. Antiinflammatory therapy with Canakinumab for atherosclerotic disease. N Engl J Med. (2017) 377:1119–31. 10.1056/NEJMoa1707914 28845751

[72] Roguin A Koch W Kastrati A Aronson D Schomig A Levy AP. Haptoglobin genotype is predictive of major adverse cardiac events in the 1-year period after percutaneous transluminal coronary angioplasty in individuals with diabetes. Diabetes Care (2003) 26:2628–31. 10.2337/diacare.26.9.2628 12941730

[73] Ruzevick J Jackson C Pradilla G Garzon-Muvdi T Tamargo RJ. Aneurysm formation in proinflammatory, transgenic haptoglobin 2-2 mice. Neurosurgery (2013) 72:70–6; discussion: 76. 10.1227/NEU.0b013e318276b306 23096414

[74] Sadrzadeh SM Bozorgmehr J. Haptoglobin phenotypes in health and disorders. Am J Clin Pathol. (2004) 121(Suppl.):S97–104. 10.1309/8GLX5798Y5XHQ0VW 15298155

[75] Schaer DJ Vinchi F Ingoglia G Tolosano E Buehler PW. Haptoglobin, hemopexin, and related defense pathways-basic science, clinical perspectives, and drug development. Front Physiol. (2014) 5:415. 10.3389/fphys.2014.00415 25389409 PMC4211382

[76] Shah SV Alam MG. Role of iron in atherosclerosis. Am J Kidney Dis. (2003) 41:S80–83. 10.1053/ajkd.2003.50091 12612959

[77] Smithies O Connell GE Dixon GH. Chromosomal rearrangements and the evolution of haptoglobin genes. Nature (1962a) 196:232–6. 13989613 10.1038/196232a0

[78] Smithies O Connell GE Dixon GH. Inheritance of haptoglobin subtypes. Am J Hum Genet. (1962b) 14:14–21. 13914473 PMC1932187

[79] Sorrentino SA Besler C Rohrer L Meyer M Heinrich K Bahlmann FH. Endothelial-vasoprotective effects of high-density lipoprotein are impaired in patients with type 2 diabetes mellitus but are improved after extended-release niacin therapy. Circulation (2010) 121:110–22. 10.1161/CIRCULATIONAHA.108.836346 20026785

[80] Sun J Zhao XQ Balu N Neradilek MB Isquith DA Yamada K. Carotid plaque lipid content and fibrous cap status predict systemic CV outcomes: the MRI substudy in AIM-HIGH. JACC Cardiovasc Imag. (2017) 10:241–9. 10.1016/j.jcmg.2016.06.017 28279371 PMC5347460

[81] Swaminathan S Fonseca VA Alam MG Shah SV. The role of iron in diabetes and its complications. Diabetes Care (2007) 30:1926–33. 10.2337/dc06-2625 17429063

[82] Tawakol A Ishai A Takx RA Figueroa AL Ali A Kaiser Y. Relation between resting amygdalar activity and cardiovascular events: a longitudinal and cohort study. Lancet (2017) 389:834–45. 10.1016/S0140-6736(16)31714-7 28088338 PMC7864285

[83] Vardi M Levy AP. Is it time to screen for the haptoglobin genotype to assess the cardiovascular risk profile and vitamin E therapy responsiveness in patients with diabetes? Curr Diab Rep. (2012) 12:274–9. 10.1007/s11892-012-0265-8 22427005 PMC3590812

[84] Wassell J. Haptoglobin: function and polymorphism. Clin Lab. (2000) 46:547–52. 11109501

[85] White HD Simes J Stewart RA Blankenberg S Barnes EH Marschner IC. Changes in lipoprotein-associated phospholipase A2 activity predict coronary events and partly account for the treatment effect of pravastatin: results from the long-term intervention with pravastatin in Ischemic disease study. J Am Heart Assoc. (2013) 2:e000360. 10.1161/JAHA.113.000360 24152981 PMC3835245

[86] Xing L Higuma T Wang Z Aguirre AD Mizuno K Takano M. Clinical significance of lipid-rich plaque detected by optical coherence tomography: A 4-year follow-up study. J Am Coll Cardiol. (2017) 69:2502–13. 10.1016/j.jacc.2017.03.556 28521888

